# Soft climbing robot with magnetic feet for multimodal locomotion

**DOI:** 10.1038/s41598-023-35667-7

**Published:** 2023-05-24

**Authors:** Gijun Park, Hugo Rodrigue

**Affiliations:** School of Mechanical Engineering, Sungkyunkwan University, Suwon, 16419 Republic of Korea

**Keywords:** Mechanical engineering, Electrical and electronic engineering

## Abstract

Inspection robots that can be used to inspect man-made structures have significant potential for industrial applications, but existing soft robots are not well suited for the exploration of complex metallic structures with many obstacles. This paper proposes a soft climbing robot well suited for such conditions as the robot uses feet with a controllable magnetic adhesion. It uses soft inflatable actuators to control this adhesion as well as the deformation of the body. The proposed robot consists of a robot body that can bend and lengthen, robot feet that can magnetically adhere to and detach from metallic surface, and rotational joints connecting each foot to the body to give the robot additional flexibility. It combines extensional soft actuators for the deformation of the body and contractile linear actuators for the robot feet, and the robot can produce complex deformations of the body that allow it to overcome a variety of scenarios. The capabilities of the proposed robot were verified through the implementation of three scenarios on metallic surfaces: crawling, climbing, and transitioning between surfaces. The robots could crawl or climb nearly interchangeably, could transition to and from horizontal surfaces to either upward or downward vertical surfaces.

## Introduction

Steel structures are used in various industrial fields ranging from civil engineering facilities to specialized equipment and vehicles such as gantry cranes, containers, bridges, railway vehicles and large construction machinery. Safety checks and maintenance work on these structures are currently entirely dependent on manpower but these structures are often very large and composed of a complex assembly of struts or panels joined together. These characteristics make the inspection of these structures time consuming, tedious and can cause injuries to the worker through repeated motions or falls. For these reasons, various types of inspection robots have been developed for these types of structures^1,2^.

Diverse types of rigid robots have been developed that can adhere to diverse surfaces using different attachment methods. Wall-climbing methods for four-legged wall-climbing robots have been developed through the use of magnets, hooks, gecko-inspired structures, and wet adhesion pads^3–7^. Approaches for four-wheeled robots capable of adhering to walls have been developed using electric duct fans or using magnets while the wheels move the robot^8,9^. Crawling robots using tracks with different adhesion mechanisms have been used such as magnets or suction pads able to perform advanced movements such as moving from the ground to the wall and overcoming obstacles^10–12^. Biomimetic inchworm-like robots with similar mechanisms have been shown to have a remarkable ability to overcome obstacles^13,14^. Robots with on-board sensing systems and batteries have demonstrated their capabilities to analyze fatigue and cracks on bridges^15,16^. However, these robots making use of motors and rigid mechanisms are bulky and lack flexibility such that they are limited to operate in very open environment such as regular and continuous surfaces with large clearances and with few obstacles.

Soft robots are inherently compliant due to the soft materials from which they are composed, and this compliance allows them to be highly adaptable to their environment^17,18^. Worm and inchworm biomimetic robots making use of friction for crawling locomotion have been developed^19–22^. But these haven’t shown to be capable of climbing walls. They can be modified for pipe climbing by using feet that wrap around the outer section of the pipe to produce a gripping force^23–26^, and through the inside of pipes by expanding their feet to create friction with the pipe^27–29^. The feet of biomimetic robots using gecko and worm-inspired gaits can be replaced by suction cups to increase adhesion and allow for the climbing of vertical surfaces such as walls^30–32^. The use of segmented and omnidirectional bending bodies has enabled the transition between ground and wall^33,34^, but this has not been demonstrated for transitioning to walls at a downward right angle from the ground. One potential issue with the use of suction cups for adhesion is that they only work on flat and untextured surfaces, which severely limits their application in real scenarios. Electrostatic adhesive crawling robots have been developed as an alternative^35–37^, but these require high voltages for operation which may not be safe in industrial environment.

This paper proposes a climbing robot using magnetic adhesion and making use of soft inflatable actuators to control the magnetic adhesion of the feet and to deform the body. This allows the robot to easily operate on magnetic surfaces and to transition between perpendicular surfaces. The robot body consists of a deformable scissor mechanism capable of being deformed linearly, horizontally, and vertically, and it uses extensional soft actuators with an initial zigzag configuration to produce extensional forces to drive the deformation of the body of the robot. It also has rotational joints at each end of the body to increase the range of motion of the robot. The design and assembly of the robot is shown followed by an assessment of the performance of the individual elements of the robot. Finally, the capabilities of the robot for crawling, climbing and for transitioning between perpendicular surfaces are demonstrated.

## Robot design

**Figure 1:**
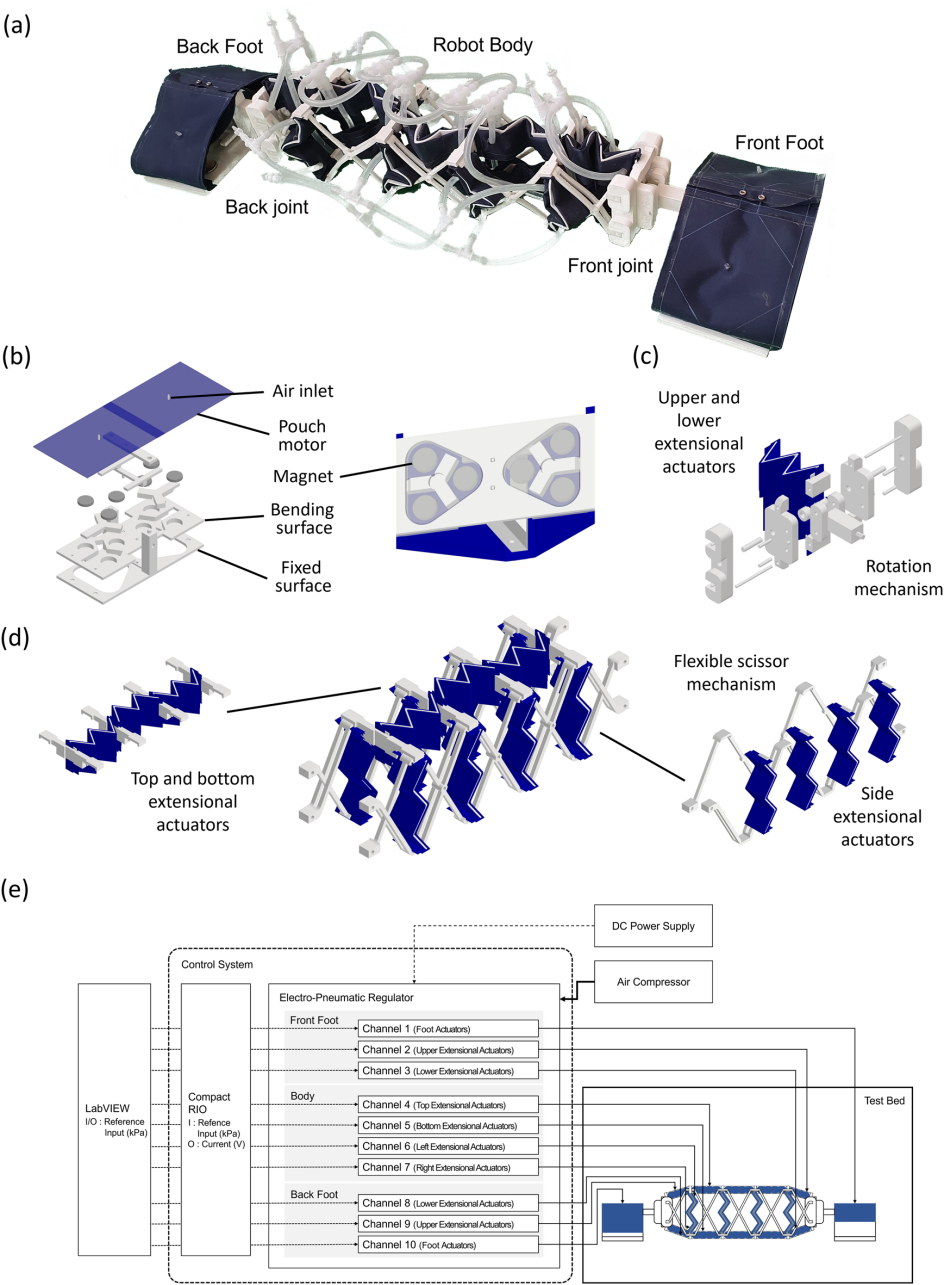
(**a**) Robot design consisting of two feet, two rotational joints and a robot body, (**b**) assembly of magnetic foot, (**c**) assembly of rotational joint, and (**d**) assembly of robot body.

A crawling soft robot with magnetic feet is proposed in this work where soft inflatable actuators are used to deform the body and to control the magnetic adhesion of the feet of the robot (Fig. 1a). The robot consists of a bending robot body, magnetic feet, and rotational joints connecting each foot to the body to give additional flexibility to the body's motion when transitioning between surfaces. The body can bend bidirectionally, contract and extend, and it is responsible for the crawling and turning motions. The rotational joints are used specifically to transition between surfaces such as between the ground and a wall. The particularity of this crawling robot is its ability to magnetically attach and detach from magnetic surfaces using soft inflatable actuators.

Each robot foot consists of two surfaces located in the same plane where one is fixed and the other can deform about its center through living hinges (Fig. 1b). The magnets are located on the deformable surface and adhesion occurs when both surfaces are along the same plane. The rotation of the surface containing the magnets causes the magnets to become detached from the ground and the feet to lose adhesion. A rigid support structure is used to connect the feet to the rotational joint and pouch motors are used to detach the magnets from the ground^38,39^. These pouch motors are made from a thermoplastic polyurethane (TPU) covered nylon technical textile which is sealed using an impulse sealer. Three neodymium magnets are attached to the left and right of the folding surface of each foot for a total of six magnets per foot. The magnets are permanent magnets that have a magnetic power of 5000 Gauss and are sufficient to maintain adhesion of the robot to magnetic surfaces in different orientations.

The rotational joints joining the feet to the body consist of a single degree of freedom (DOF) joint with a rigid rotation axis that can rotate in the vertical direction (Fig. 1c). Actuation of this joint is achieved through the pressurization of extensional soft actuators installed between the joint and the body. Although the body can bend in the same direction as the joint, the body produces a smooth and rather limited bending deformation while the rotational joints produce a deformation around a defined axis at each end of the robot. This motion is necessary for the ends of the robot to have the ability to search for adhesion points while maintaining the adhesion of the robot's feet when transitioning between perpendicular surfaces.

The robot body consists of a flexible scissor mechanism with extensional soft actuators used to produce the contraction and extension of the scissor mechanism (Fig. 1d). Scissor mechanisms are generally used to produce rigid linear deformations along a single axe, but the scissor mechanism used in the robot is made from 3D printed TPU parts whose flexibility allows the mechanism to bend laterally in both the horizontal and vertical directions. Extensional soft actuators are positioned on each of the four sides of the mechanism such that the actuators on the top and bottom parts of the robot bend the robot in the opposite direction, and those on the sides of the robot cause the robot to bend towards the pressurized actuators. The extension of the body is caused by simultaneous actuation of the top and bottom actuators, and its contraction by simultaneous actuation of the side actuators.

As mentioned previously, the deformation of the scissor mechanism and the rotational joints is realized through extensional soft actuators which consist of two inflatable tubes placed on both sides of a zigzag-shaped flexible structure. The inflatable tubes are made from the same material as the pouch motor and the zigzag-shaped flexible structure made from the same TPU material as the scissor mechanism. The inflatable tubes are initially bent following the shape of the zigzag-shaped flexible structure and inflate into a straight position upon pressurization which produces an extensive force between both ends of the actuator. Deflation of the tubes causes the flexible structure to push back the tubes into their original position.

Pneumatic control of the actuators is performed using a real-time controller (CompactRIO, NI), two electro-pneumatic regulators (ITV2030, SMC) with eight pneumatic channels each and an external pneumatic pump to supply the air pressure (Fig. 1e). Ten of the sixteen channels are used to control the robot where two are used for the front and back foot, four for the upper and lower extensional actuators of the front and back foot, two for the top and bottom extensional actuators of the body, and two for the left and right extensional actuators of the body.

## Experimental results

### Magnetic feet characteristics

**Figure 2:**
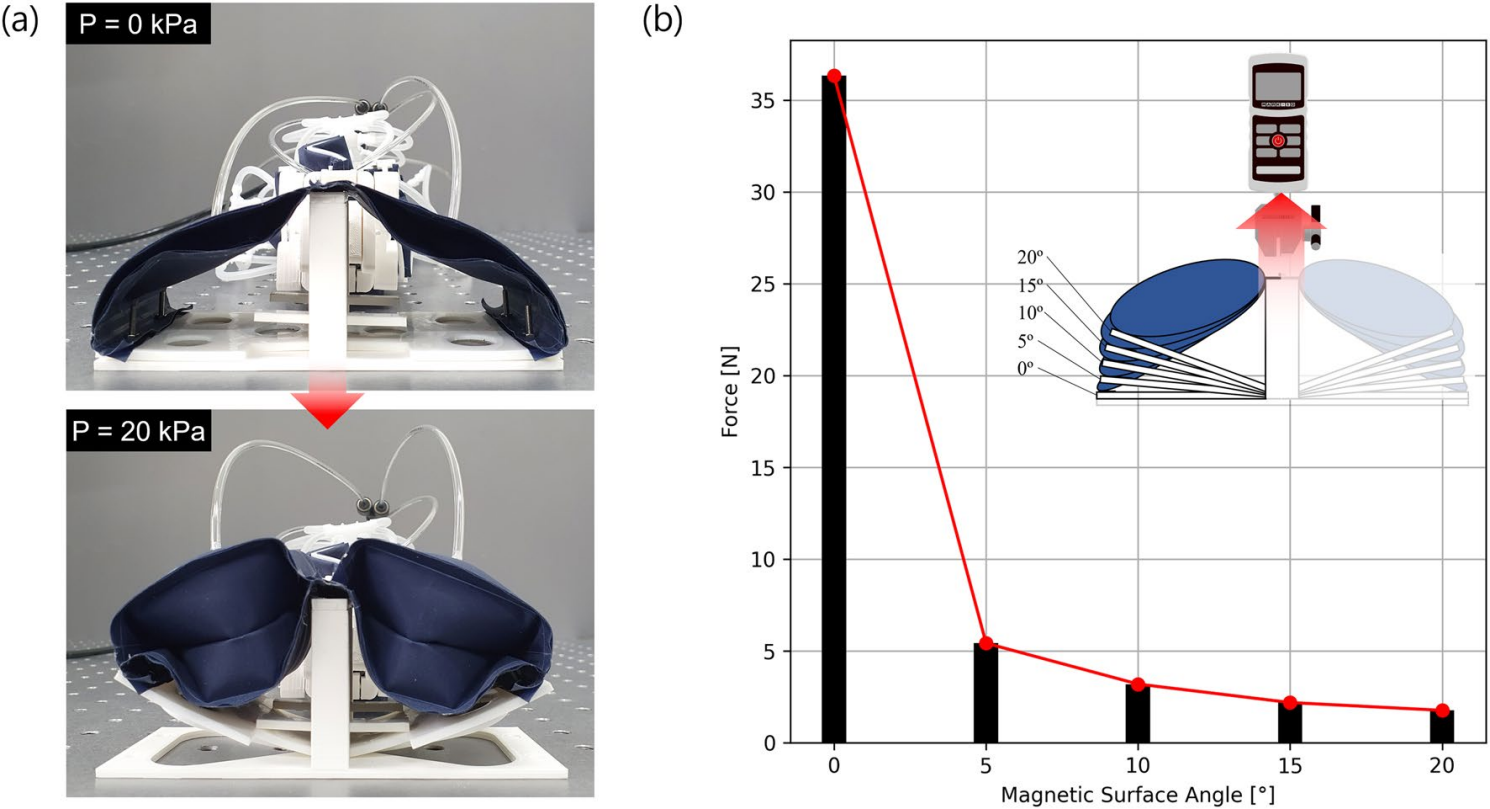
(**a**) Deformation of the feet for the adhesion test, and (**b**) adhesion force test results for the feet at different magnetic surface angles.

The process of attaching and detaching the magnetic feet from the contact surface is necessary to produce the anchoring-based locomotion typical of inchworm robots. The feet adhere to ferromagnetic surfaces using the permanent magnets located on each foot and pouch motors are used to detach the surface containing the magnets from the contact surface. When inflated, the pouch motors expand laterally and contract along their length while producing a force which rotates the free ends of the bottom surface containing the magnets (Fig. 2a). Once detached from the ferromagnetic surface, the magnets produce only a weak magnetic force. When the pouch motors are depressurized, the bottom surface containing the magnets unbends towards the ferromagnetic surface due to the force produced by the magnets.

The adhesive force of the robot foot at different angles was measured by varying the magnetic surface angle using a physical angle limiter and actuating the pouch motors. Then, the foot assembly is pulled from the magnetic surface using a linear tensile testing machine (ESM-750, MARK-10) equipped with a 100 N force gauge (M5-20, MARK-10) and a linear speed of 40 mm/mins. A force of 36 N is required to detach the feet when the magnetic surface is at an angle of 0° (Fig. 2b). Changing the magnetic surface angle even slightly significantly reduces the adhesion force which reduces to 5.42 N at an angle of 5° and 1.76 N at an angle of 20°. This force is sufficient to maintain the feet on non-horizontal surfaces yet allowing them to slide along the surface while the other foot remains in a fixed position.

### Extensional soft actuator characteristics

**Figure 3:**
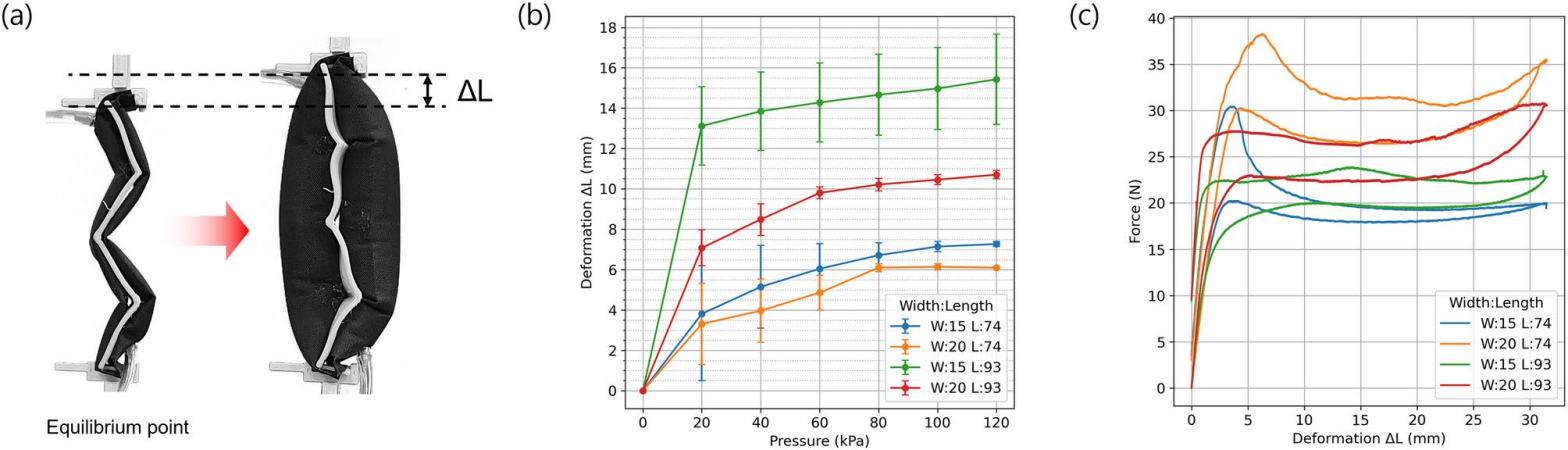
(**a**) Displacement of the extensional soft actuator, (**b**) the extension deformation versus pressure for different actuator dimensions, and (**c**) the force versus deformation for different actuator dimensions at a pressure of 120 kPa.

The deformation of the robot body occurs through the inflation of extensional soft actuators. These soft actuators consist of two inflatable tubes placed on both sides of a zigzag-shaped deformable structure which unfolds upon pressurization of the tubes (Fig. 3a). Three dimensions of these actuators are used in the robot and the different dimensions and locations where they are used are shown in Table 1. The extension deformation from equilibrium of the different actuators was tested without any payloads by using a marker with the displacement recorded using a motion tracking device (Optitrack, V120:Trio). All dimensions exhibited a large deformation even at pressures of 20 kPa and then a slight increase as the pressure is increased to 120 kPa (Fig. 3b). The tubes are simultaneously becoming straight which increases their length along the axis of the actuator and shrinking along the tube’s length due to the rounding of their structure. Thus, actuators with wider tubes were seen to extend less than the ones with thinner tubes as the wider tubes shrink along their length due to the more pronounced rounding of their structure. Also compared to 20 mm wide actuators, actuators with a width of 15 mm show a significant larger error range particularly at lower pressures. This is because the pressure starts to deform the skeleton which resists the expansion and may cause lateral deformations resulting in larger variations in the early part of the deformation. The force versus displacement behavior of each actuator dimension was measured from their respective equilibrium point (Fig. 3c), and the actuators produced a relatively steady force throughout the motion. Wider actuators were able to produce a higher peak force for the same actuator length while longer actuators produced a slightly smaller force than shorter ones due to the tubes more easily deforming laterally.

**Table 1:** Dimensions of extensional actuators at different locations on the robot.

Actuator location	Dimensions	
	Length (mm)	Width (mm)
Side of body	74	15
Top and bottom of body	74	20
Joint actuator	93	20

### Body deformation characteristics

The flexible scissor mechanism in combination with the extensional soft actuators is what enables the deformation of the robot. The forward crawling locomotion in either the horizontal or vertical direction is achieved by alternately extending and contracting the body. Extension of the body is achieved by simultaneously pressurizing the top and bottom extensional soft actuators to extend the robot body with the side actuators depressurized, and contraction of the body by pressurizing the side actuators with the top and bottom actuators depressurized.

**Figure 4:**
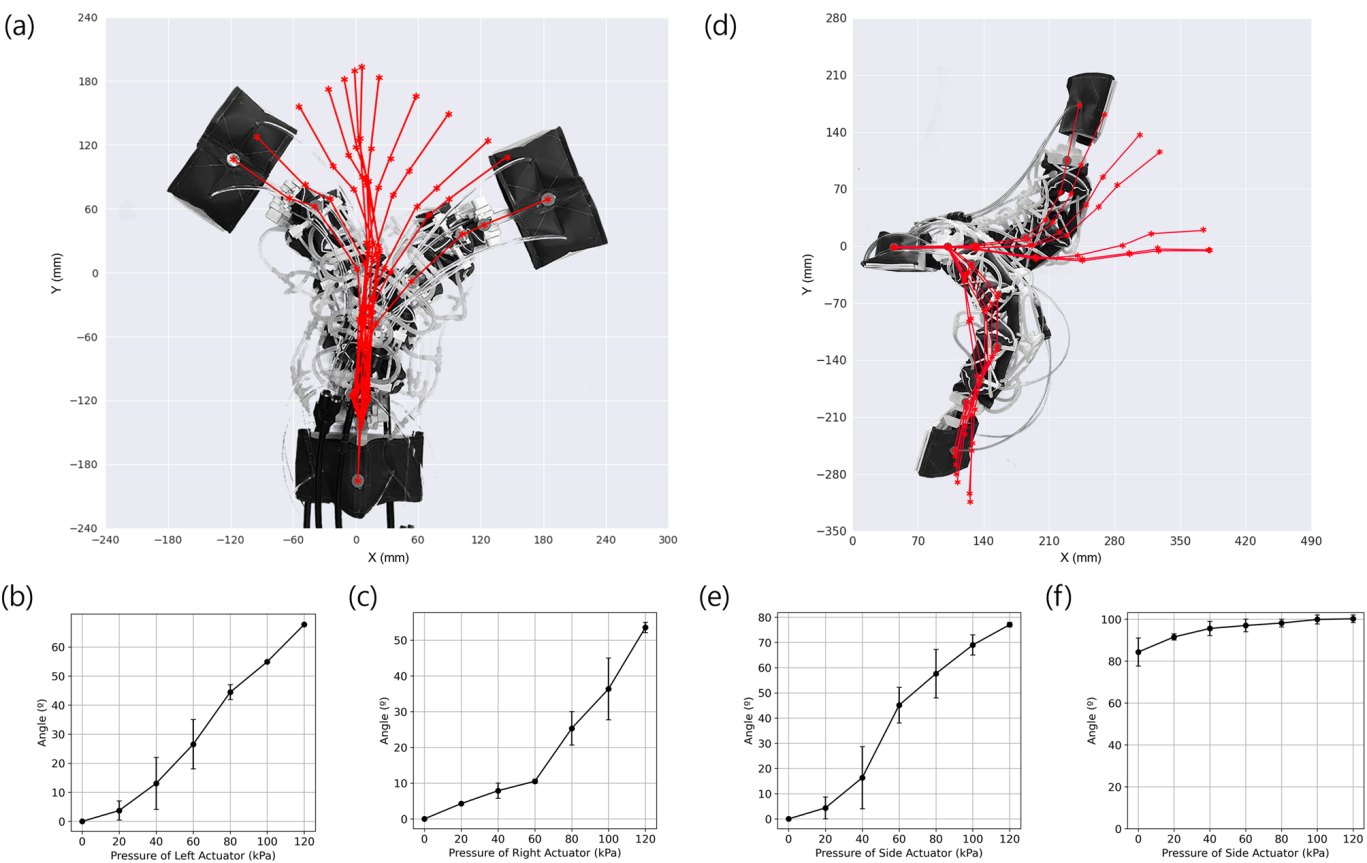
(**a**) Lateral range of motion of the robot when placed in the horizontal plane. Bending angle versus pressure of the body of the robot when (**b**) bending to the left and (**c**) bending to the right. (**d**) Range of motion of the robot in the vertical plane, (**e**) bending angle versus pressure when bending upwards and (**f**) downwards.

Lateral bending of the body is achieved by pressurizing the actuators on the side that the robot is made to bend towards. In the horizontal position, the angle between the two ends of the robots was measured for different values of pressures in the actuators (Fig. 4a). In can be seen that the angle changes relatively linearly with an increase in pressure as the body of the robot offers a resistance that increases with its displacement (Fig. 4b,c). Vertical bending of the body is achieved by pressurizing the actuators on the opposite side that the robot is made to bend towards (Fig. 4d). First, the robot was made to bend upwards from the horizontal position by inflating the bottom actuators (Fig. 4e). In this case, sufficient pressure is necessary to lift the free end of the robot and it reaches an angle of 78° at a pressure of 120 kPa. Bending down can be done using only gravity, but the top actuators can be used to produce a rounded shape that can help in clearing corners when transitioning between parallel surfaces. The maximum angle reached while bending downwards is 102° at a pressure of 120 kPa (Fig. 4f). Some angular deformation error can be seen for most results which is likely caused by friction in the body, the nonlinearities of the actuator themselves, and the general nonlinearities of the deformable scissor mechanism of the robot body. The angle values of the previous experiments were measured using markers on the body and the feet and using trigonometric functions to extract the angle by using a motion tracking device (Optitrack, V120:Trio).

### Rotational joint characteristics

**Figure 5:**
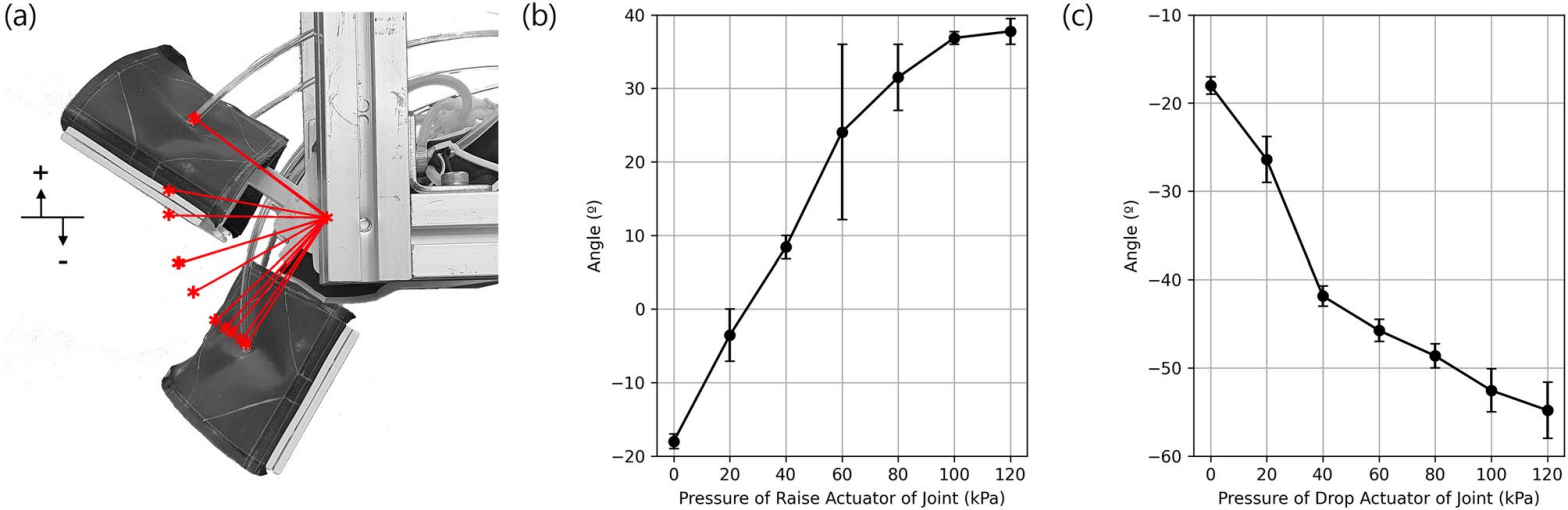
(**a**) Deformation of the rotational joint, (**b**) rotation angle versus pressure when rotating upward and (**c**) downwards.

The rotational joints connecting the body to the feet can produce a concentrated deformation at each end of the robot (Fig. 5a). This joint contains upper and lower extensional actuators located on the upper and lower portions of the joint. Rotating the joint upwards requires the pressurization of the lower actuator and the joint is initially at a negative angle from the horizontal when the actuators are unpressurized. The angle of the joint was measured using a marker and motion tracking device as done previously. Pressurizing the lower actuator allows the joint to reach its maximum upward angle of 38° at a pressure of 120 kPa (Fig. 5b). In this case, there is a particularly large error range at 60 to 80 kPa which is due to the nonlinearities of the actuator used in the joint. This maximum angle is due to the mechanical limit of the joint design. The downward rotation requires pressurizing the upper actuator and the joint in this direction can reach an angle of -55° at a pressure of 120 kPa (Fig. 5c). This range of motion is sufficient to help the robot overcome a wide range of obstacles.

## Robot locomotion

**Figure 6:**
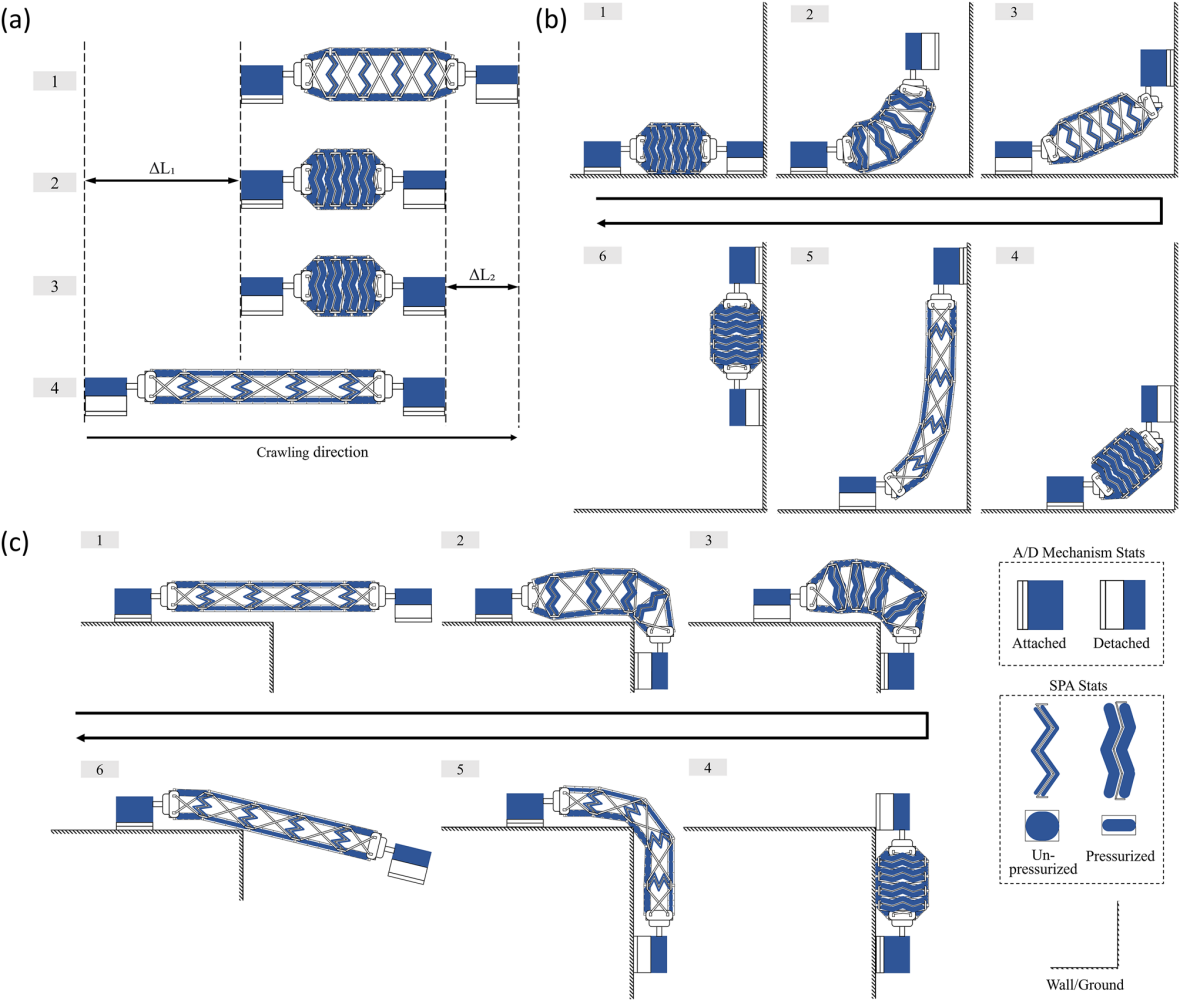
(**a**) Steps for crawling or climbing locomotion, (**b**) steps for transition from a horizontal surface to an upward vertical surface, and (**c**) steps for a transition from a horizontal surface to a downward vertical surface.

The proposed robot is capable of crawling horizontally, climbing vertically, and transitioning from a horizontal surface to either an upward or downward perpendicular surface. The robot uses a two-anchor crawling mode of locomotion and, by magnetically anchoring one of its feet, can deform the body in any direction. As the body is stiff enough to prevent it from deforming under its own weight, the crawling locomotion is the same whether crawling on a flat surface or up a wall. Thus, crawling horizontally or vertically is done by anchoring the back foot, expanding the body, anchoring the front foot, contracting the body, and repeating this process (Fig. 6a).

Transferring between a horizontal surface and an upward perpendicular surface involves the robot crawling close to the transition edge between surfaces with the body contracted, anchoring the back foot and using a combination of the rotational joints and flexible scissor mechanism deformations to extend the body and bring the front foot perpendicular onto the second surface, then additional cycles of extension and contraction may be needed before being able to anchor both feet on the second surface (Fig. 6b). The additional cycles of contraction and extension may be necessary as the stride of the robot is less than the body length and a single cycle is not enough to bring the entire length of the robot onto the second surface. Transferring between a horizontal surface and a downward perpendicular surface involves a process very similar to that of the upward surface (Fig. 6c). Cycles of anchoring the back foot, extending the body, anchoring the front foot, and contracting the body are used to move the feet forward at each cycle until both feet can transition onto the vertical surface (Supplementary Video 1).

### Crawling

**Figure 7:**
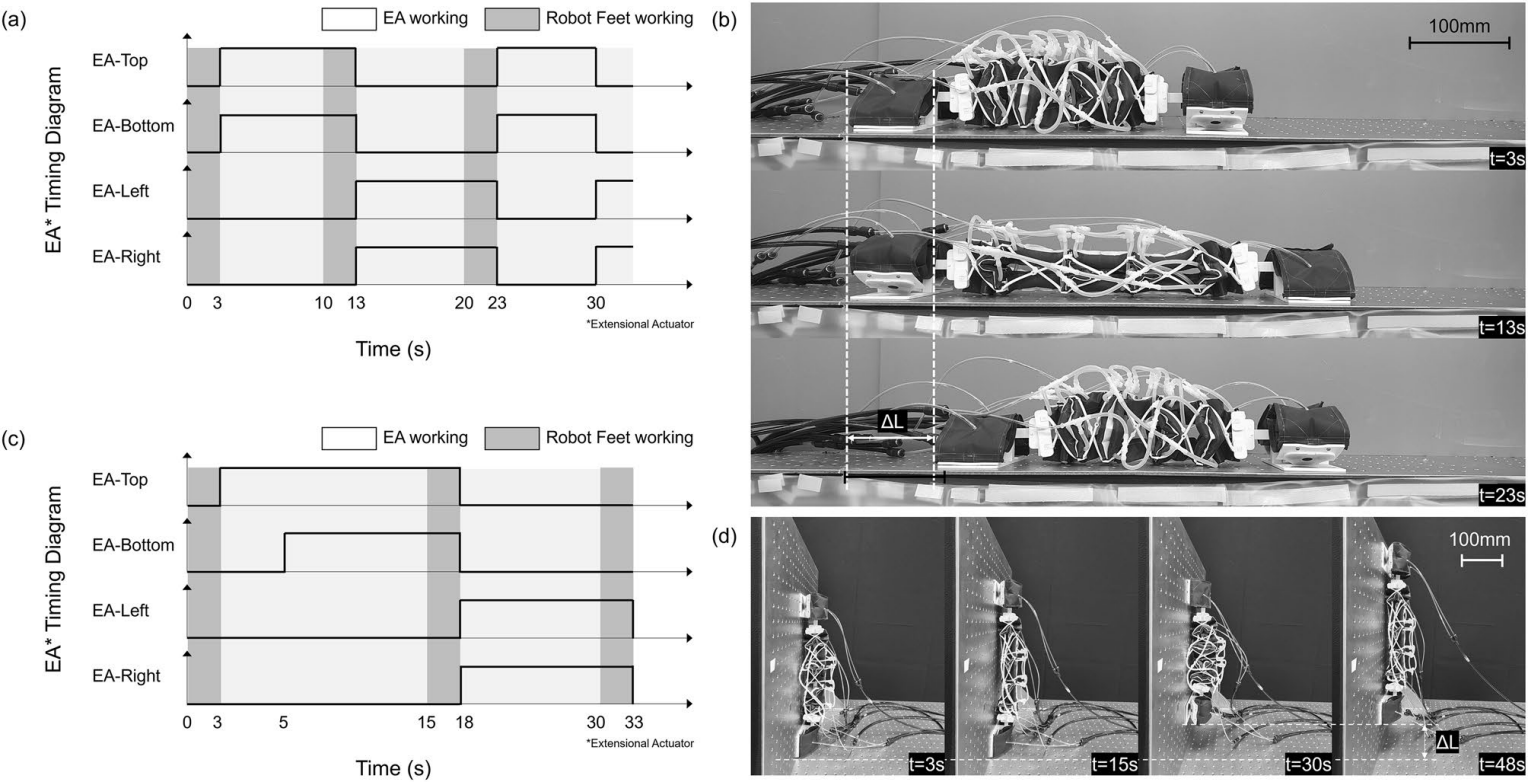
(**a**) Pressure patterns and (**b**) movement during the crawling locomotion. (**c**) Pressure patterns and (**d**) movement during the climbing locomotion.

First, the crawling locomotion ability of the proposed robot was tested on a flat surface where the extension of the body was realized by simultaneously pressurizing the top and bottom extensional actuators (Fig. 7a). For this scenario, the pouch motor used to detach the feet used a pressure of 20 kPa and the extensional soft actuators used for the contraction and inflation mechanism of the body used a pressure of 120 kPa. The stride length of the robot was 8.14 cm, which corresponds to 27.1% of body lengths per stride, and the average speed of the robot was 5.31 mm/s (Fig. 7b). It would be possible to adjust the stride length of the robot by using a lower pressure to produce precise robot displacements and a higher flow rate in and out of the actuators could be used to further increase the speed of the robot.

### Climbing

The climbing locomotion scenario was implemented in the vertical axis with gravity acting along the length of the robot. Similar pressures were used as in the horizontal crawling scenario, but the timing of the actuation of the extension of the body was changed so that the top actuators of the robot body extend first to maintain contact with the surface (Fig. 7c). The average displacement per stride was approximately 8.08 cm (Fig. 7d), which is slightly less than for the horizontal crawling locomotion. This is due to gravity which acts against the extension of the robot. Both crawling and climbing locomotion were also tested in wet conditions (Supplementary Video 2).

### Transitioning between surfaces

**Figure 8:**
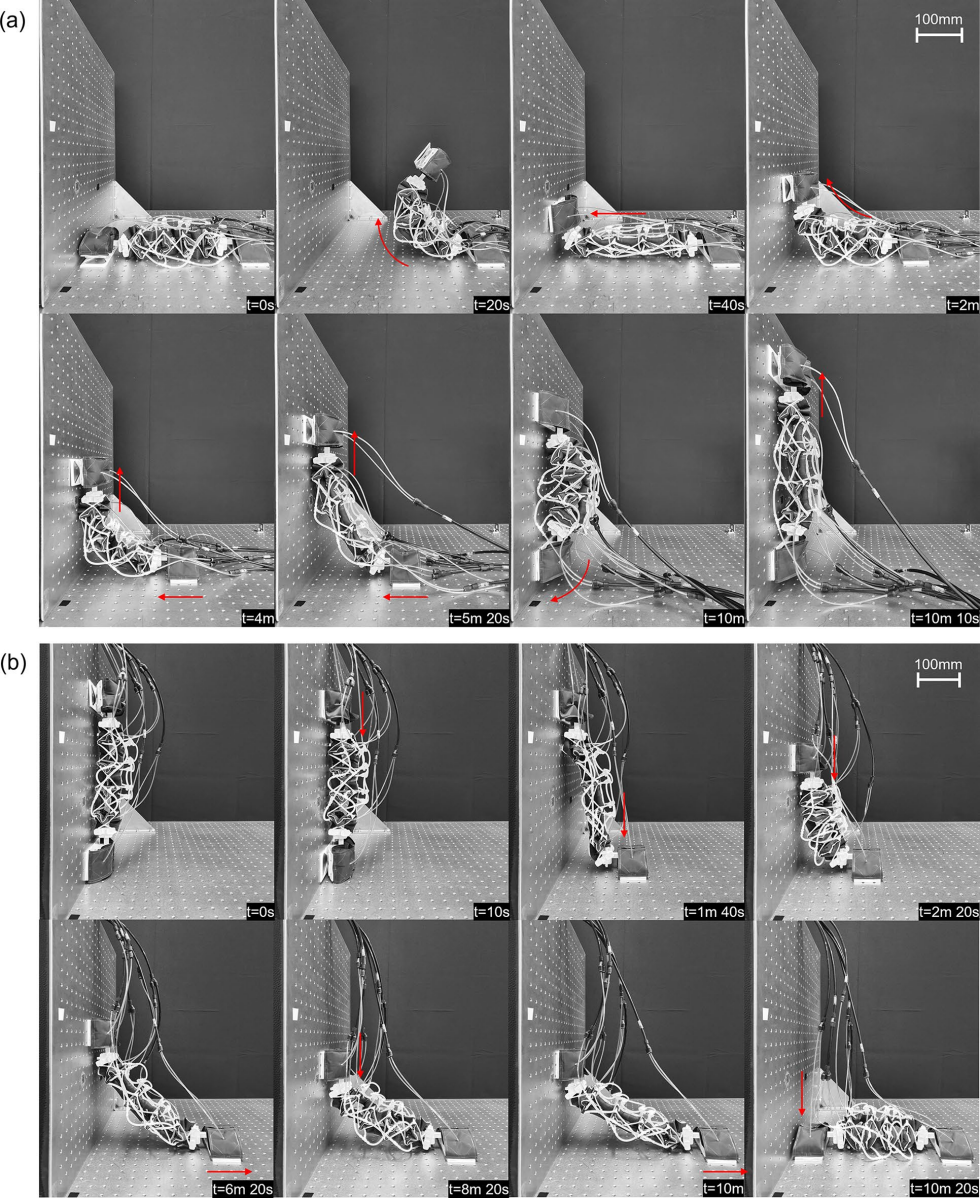
Demonstration of transitioning from (**a**) the ground to an upward vertical wall, and (**b**) the opposite transition.

The robot can transition from a horizontal plane to a vertical one at a perpendicular angle using the previously detailed process (Fig. 8a). Pressures of 80 kPa are used in the rotational joints near the back and front feet to bend the ends of the robot, pressures of 120 kPa are used in the actuators on the bottom of the robot body to arc the body upwards, and pressures of 120 kPa are used for the contraction and extension of the robot body. It is to be noted that only the actuators on the bottom of the robot body are used for extension of the body, and that these are deflated while pressurizing the actuators on the side of the robot body during contraction. During the first step, the robot body’s back foot is fixed, and the front foot is lifted from the surface. A total of 6 cycles were necessary to be able to transition both feet onto the vertical wall. The reverse transition is done by following the inverse series of steps using a total of 4 cycles (Fig. 8b).

**Figure 9:**
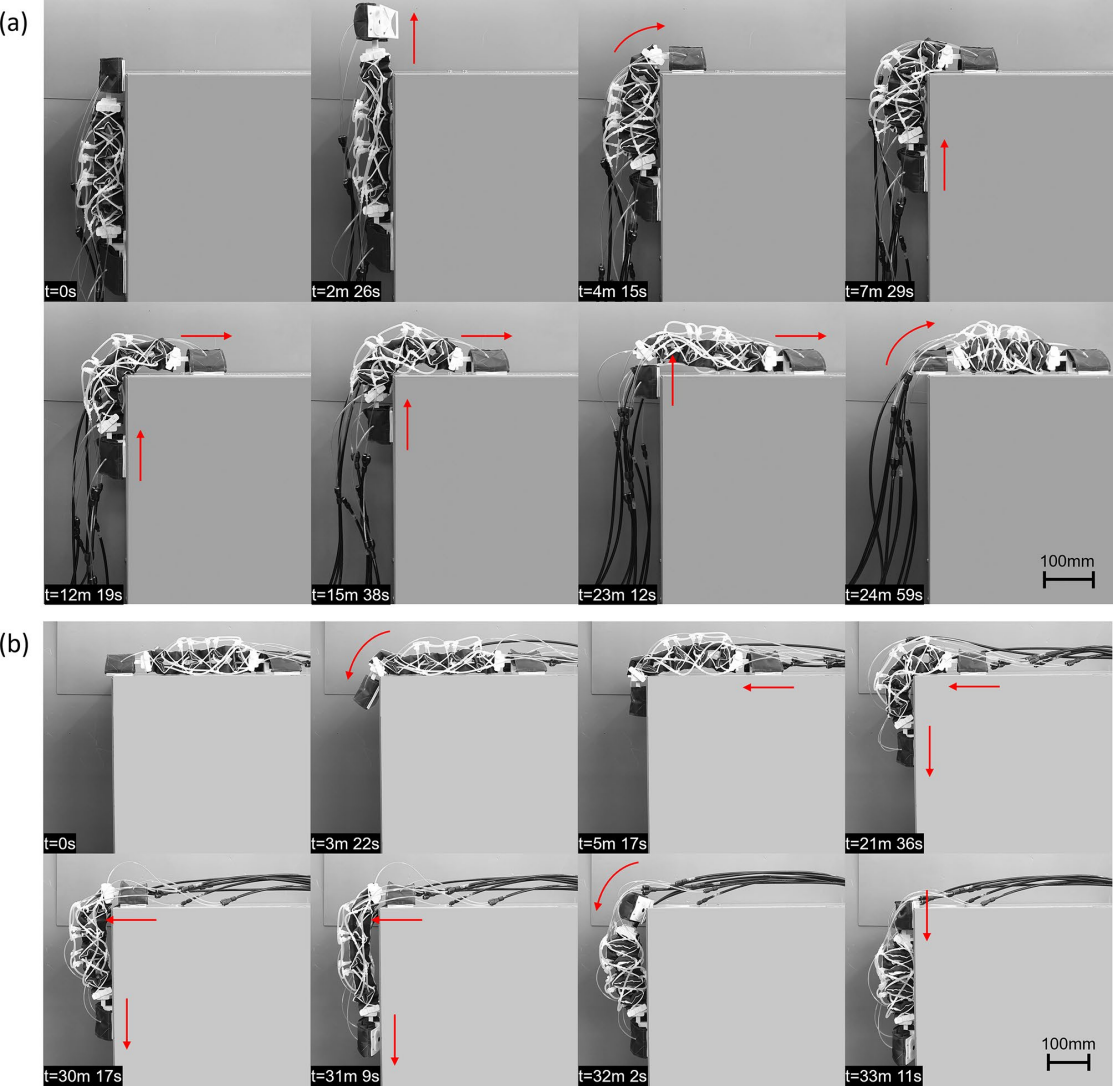
Demonstration of transitioning from (**a**) a vertical wall to a horizontal surface above the robot, and (**b**) the reverse transition.

The robot can transition from a vertical plane to a horizontal plate located above it (Fig. 9a). First, the front of the body is moved onto the perpendicular surface by detaching the front foot, extending the body, bending the front foot’s joint and attaching the front. Then the steps of detaching the back foot, contracting the body, attaching of the back foot, detaching the front foot and extension of the body are repeated until the back foot is close to the edge. At this stage, the next contraction step can be used to bring the back foot onto the horizontal surface to finish the transition. During the transition, the robot body’s top actuators are used for extension of the robot body to maintain the feet attached to the ground despite the force of the surface’s corner. Similar pressures as the previous test were used. A total of 9 cycles were necessary to transition between the two surfaces. The reverse transition is done by following the inverse series of steps using a total of 11 cycles (Fig. 9b).

## Conclusion

This paper proposed a climbing robot using magnetic adhesion and making use of soft inflatable actuators to control the magnetic adhesion of the feet and to deform the body. This allows the robot to produce multimodal surface transitions between metallic surfaces. The proposed robot can adjust the adhesive force of the feet using pouch motors in order to attach and detach the feet while the robot’s body can bend horizontally and vertically as well as contract and extend. Additional joints are located between the feet and the body to enable the robot to transition between surfaces more easily.

The capabilities of the proposed robot were verified through the demonstration of three scenarios: crawling, climbing, and transitioning between surfaces. The robot was able to climb with nearly the same stride length as when crawling horizontally. The robot had to repeat multiple cycles of contraction and extension to transfer between surfaces. The easiest part of this transition was transferring from a horizontal surface to an upward vertical surface which required 6 cycles. The transition from a horizontal surface to a downward vertical surface took the most cycles with a total of 11. A robot body with a higher stride length will be necessary to enable more rapid transitions between surfaces.

The proposed robot is meant to function solely on metallic surfaces. This means that the robot is not significantly affected by the surface condition and can exert strong adhesive forces if the proper type of surface is encountered. Future work will focus on the integration of larger diameter actuators with higher stroke to use lower pressures within the robot body and on the integration of a pneumatic system for untethered application.

## Supplementary Information

Supplementary Legends.

Supplementary Video 1.

Supplementary Video 2.

## Data Availability

The datasets generated during and/or analyzed during the current study are available from the corresponding author on reasonable request.
