# Supplementary material for: Soft climbing robot with magnetic feet for multimodal locomotion
Source: Sci Rep. 2023 May 24;13:8377. doi: 10.1038/s41598-023-35667-7 (PMC10209148; doi:10.1038/s41598-023-35667-7)
Supplement: Supplementary file 1 — Supplementary Legends. [file 41598_2023_35667_MOESM1_ESM.docx]

Supplementary Video 1. Multimodal locomotion of the soft climbing robot with magnetic feet.
